# Supplementary material for: Causal Relationship between Aging and Anorexia Nervosa: A White-Matter-Microstructure-Mediated Mendelian Randomization Analysis
Source: Biomedicines. 2024 Aug 16;12(8):1874. doi: 10.3390/biomedicines12081874 (PMC11351342; doi:10.3390/biomedicines12081874)
Supplement: Supplementary file 1 [file biomedicines-12-01874-s001.zip › Supplementary Figure/Figure S4/Singe SNP plot of MR analysis for TL to ubm-b-1464.pdf]

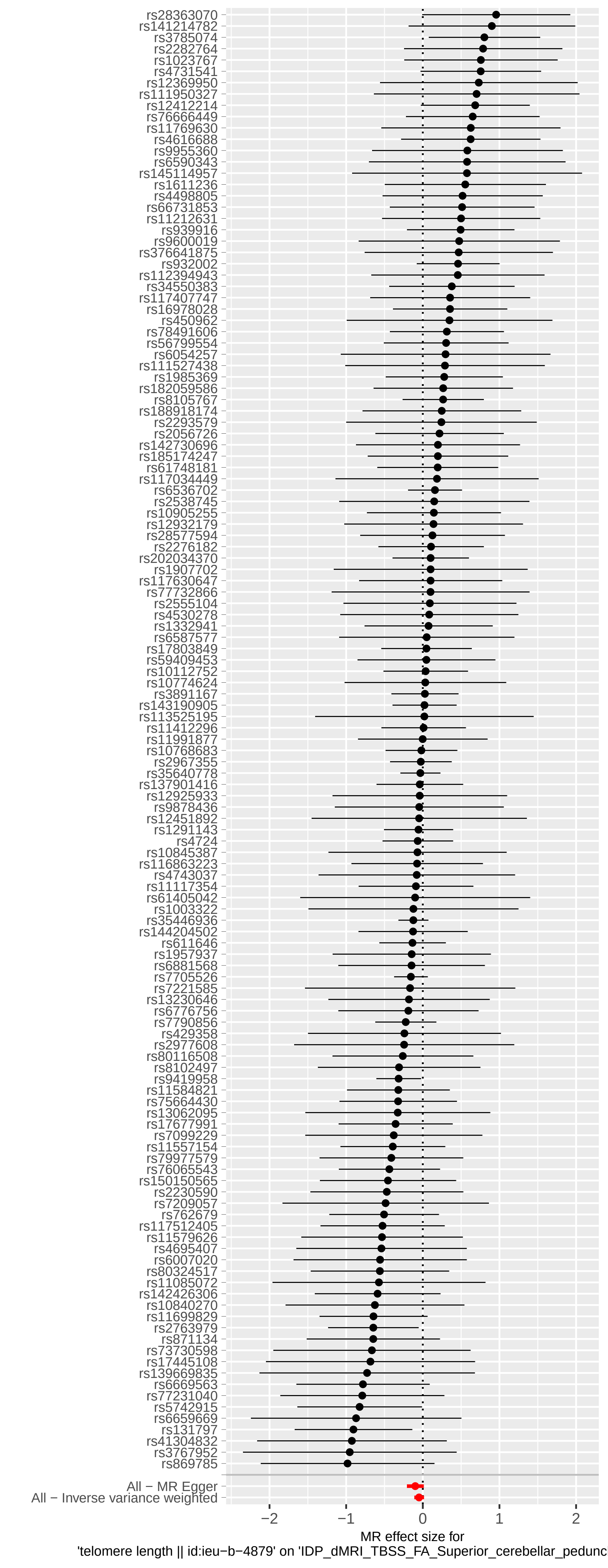
rs28363070
rs141214782
rs3785074
rs2282764
rs1023767
rs4731541
rs12369950
rs111950327
rs12412214
rs76666449
rs11769630
rs4616688
rs9955360
rs6590343
rs145114957
rs1611236
rs4498805
rs66731853
rs11212631
rs939916
rs9600019
rs376641875
rs932002
rs112394943
rs34550383
rs117407747
rs16978028
rs450962
rs78491606
rs56799554
rs6054257
rs111527438
rs1985369
rs182059586
rs8105767
rs188918174
rs2293579
rs2056726
rs142730696
rs185174247
rs61748181
rs117034449
rs6536702
rs2538745
rs10905255
rs12932179
rs28577594
rs2276182
rs202034370
rs1907702
rs117630647
rs77732866
rs2555104
rs4530278
rs1332941
rs6587577
rs17803849
rs59409453
rs10112752
rs10774624
rs3891167
rs143190905
rs113525195
rs11412296
rs11991877
rs10768683
rs2967355
rs35640778
rs137901416
rs12925933
rs9878436
rs12451892
rs1291143
rs4724
rs10845387
rs116863223
rs4743037
rs11117354
rs61405042
rs1003322
rs35446936
rs144204502
rs611646
rs1957937
rs6881568
rs7705526
rs7221585
rs13230646
rs6776756
rs7790856
rs429358
rs2977608
rs80116508
rs8102497
rs9419958
rs11584821
rs75664430
rs13062095
rs17677991
rs7099229
rs11557154
rs79977579
rs76065543
rs150150565
rs2230590
rs7209057
rs762679
rs117512405
rs11579626
rs4695407
rs6007020
rs80324517
rs11085072
rs142426306
rs10840270
rs11699829
rs2763979
rs871134
rs73730598
rs17445108
rs139669835
rs6669563
rs77231040
rs5742915
rs6659669
rs131797
rs41304832
rs3767952
rs869785
All − MR Egger
All − Inverse variance weighted
−2
−1
0
1
2
MR effect size for
'telomere length || id:ieu−b−4879' on 'IDP_dMRI_TBSS_FA_Superior_cerebellar_pedunc
